# Association between HALP score and clinical outcome in patients with aneurysmal subarachnoid hemorrhage: insights from a large cohort study

**DOI:** 10.3389/fneur.2025.1667743

**Published:** 2025-09-05

**Authors:** Jun Yang, Bingcheng Zhu, Jizong Zhao, Xiaolin Chen

**Affiliations:** ^1^Department of Critical Care Medicine, Beijing Tiantan Hospital, Capital Medical University, Beijing, China; ^2^Department of Neurosurgery, Beijing Tiantan Hospital, Capital Medical University, Beijing, China

**Keywords:** aneurysmal subarachnoid hemorrhage, HALP, prognosis, inflammation, nutrition

## Abstract

**Background:**

The HALP (hemoglobin, albumin, lymphocyte, and platelet) score is a newly emerging index for reflecting the systemic inflammation and nutritional status of patients. Previous studies have identified that HALP score is associated with clinical outcomes of various diseases. This study aims to explore the relationship between HALP score and clinical outcomes in patients with aneurysmal subarachnoid hemorrhage (aSAH).

**Methods:**

A total of 748 aSAH patients were included in this study. Patients were divided into tertiles based on their HALP score levels. At 90 days after discharge, patients received follow up and the modified Rankin Scale (mRS) was used to assess the functional outcome. Unfavorable functional outcome was defined as mRS ≥ 3. Multivariate logistic regression for poor functional outcome and postoperative complications was performed to explore the association between HALP score and clinical outcomes of aSAH patients, with adjustment for age, sex, and other important factors.

**Results:**

Compared to patients with a high HALP score, patients with a low HALP score tended to be female, had a higher Hunt Hess score, and had higher levels of glucose and creatine. After adjusting several potential confounding factors, multivariate logistic regression showed that patients with a low HALP score had a higher risk of unfavorable functional outcome (OR, 0.095, 95%CI: 0.056–0.163, *p* < 0.001). The receiver operating curve (ROC) analysis demonstrated that the area under the curve (AUC) was 0.795. Furthermore, multivariate logistic regression also demonstrated that low HALP score might increase the risk of postoperative pneumonia (OR, 0.586, 95%CI: 0.388–0.887, *p* = 0.012).

**Conclusion:**

Given that HALP score may contribute to identify aSAH patients at high risk for poor prognosis, these findings hold significant clinical relevance.

## Introduction

Aneurysmal subarachnoid hemorrhage (aSAH) is a critical medical condition occurring in 2–16 patients per 100,000 per annual worldwide. The mortality rate was estimated to be exceed 30% and only one-third aSAH patients may return to normal life after receiving optimal medical care (1). According to Zhang et al., reliable prognostic predictive factors can help to recognize aSAH patients who are at high risk for poor prognosis and facilitate the development of appropriate treatment strategies (2). Therefore, the identification of accurate and reliable prognostic markers has become a major focus of current research.

HALP score, calculated by hemoglobin, albumin, lymphocyte, and platelet, has been recognized as a straightforward and accurate index reflecting the immune, inflammation and nutritional status of patients (3). Numerous studies suggest that that inflammation may play an important role in the pathophysiological process following aneurysm rupture, which significantly influencing recovery of aSAH patients (4). Additionally, poor nutritional status is also considered as an independent risk factor of adverse prognosis and postoperative complications in aSAH patients (5, 6). Based on these findings, we hypothesize that HALP may be associated with the recovery of aSAH. However, no investigations have established whether HALP correlates with aSAH prognosis.

Therefore, the objective of this study is to investigate the relationship between the prognosis of aSAH and explore whether HALP can play an important role in prognostic predicting.

## Methods

### Patients

Data of aSAH patients were gathered from the Long-term Prognosis of Emergency Aneurysmal Subarachnoid Hemorrhage (LongTEAM) Registry study (Registration No. NCT04785976). This observational cohort investigations was conducted at Beijing Tiantan Hospital in China from January 2015 to September 2022. All patients received a brain computed tomography (CT) scan, CT angiography (CTA) or digital subtraction angiography (DSA) to confirm the diagnosis of aSAH. The criteria for inclusion were: (1) older than 18 years; (2) presence of single aneurysm; (3) treated with surgical clipping or endovascular coiling within 72 h of onset; (4) admission through the emergency department; (5) completing follow-up. The criteria for exclusion included: (1) a prior history of SAH or other neurosurgical disease; (2) previous craniotomy or intracranial vascular interventions; (3) physical disability; (4) absence of data, laboratory tests, and radiological information; (5) patients with active or chronic inflammatory disease; (6) receipt of immunosuppressive therapy in the 6 months before admission. Informed consent was secured from patients or their authorized representatives prior to enrollment.

### Data collection

Demographic data encompassed age, gender, current smoking, current drinking, diabetes, hypertension, history of heart disease, history of antiplatelet, history of anticoagulant. The preoperative clinical status included the World Federation of Neurosurgical Societies (WFNS) grade, modified Fisher Scale (mFS) grade, Graeb score, Subarachnoid Hemorrhage Early Brain Edema Score (SEBES), Glasgow Coma Scale (GCS), Hunt Hess score, intraventricular hemorrhage (IVH), preoperative hydrocephalus, loss of conscious. Max diameter of aneurysm and treatment modality were also collected. The postoperative complications included major adverse cardiovascular events (MACE), delayed cerebral ischemia (DCI), postoperative intracranial infection, postoperative stress ulcer bleeding, anemia, pneumonia, and deep vein thrombosis (DVT). The detailed diagnostic criteria for these postoperative complications were shown in Supplementary Table 1. Fasting blood samples were collected within 24 h of admission after aSAH onset. The laboratory test contained glucose, Crea, aspartate aminotransferase (AST), alanine aminotransferase (ALT), total protein (TP), albumin, globulin, white blood cell (Wbc), monocyte, neutrophil, red blood cell (Rbc), hemoglobin, platelet and lymphocyte. The normal tests value range were shown in Supplementary Table 2. Some inflammation and nutritional markers, such as neutrophil to lymphocyte rate (NLR), systemic inflammation response index (SIRI), and prognostic nutritional index (PNI) were also collected. The following equations were applied to calculate these markers: HALP = Albumin (g/L) * Hemoglobin (g/L)* Lymphocyte (10^9^/L)/Platelet (10^9^/L), NLR = neutrophils/lymphocytes, SIRI = neutrophils × monocytes/lymphocytes, and PNI = Albumin (g/L) + Lymphocyte (10^9^/L) * 5 (7, 8).

### Outcome assessment

The modified Rankin Scale (mRS) is a widely recognized neurological outcome assessment tool that evaluates functional outcomes in patients. This scale assigns scores ranging from 0, indicating the absence of any symptoms, to 6, which signifies the death of the patient. In this investigation, mRS was utilized to evaluate the clinical outcome of patients. At 90 days after discharge, patients would receive follow up via telephone or an outpatient appointment. Unfavorable outcome was defined as an mRS score from 3 to 6.

### Statistical analysis

Normally distributed variables were presented as the means ± standard deviation (SD), while variables with skewed distribution were expressed as medians (25–75th percentile). Based on the tertiles of HALP, aSAH patients were divided into 3 groups (HALP < 30.286, 30.286 ≤ HALP ≤ 43.201, and HALP > 43.201). The Cochran–Armitage test was applied to assess the trends in categorical data, whereas one-way ANOVA was employed for continuous data. The relationship between HALP and clinical outcome in aSAH patients was analyzed through multivariate logistic regression analysis. Three adjusted models were established to exclude the effects of confounding factors. In the crude model, age and sex were adjusted as confounders. The minimally adjusted model included crude model, GCS, WFNS, Hunt Hess score, loss of consciousness, pneumonia, and MACE. The fully adjusted model accounted for minimally adjusted model, Wbc, monocyte, glucose, and Crea. Additionally, we also established restricted cubic spline (RCS) to evaluate the dose–response relationships between HALP and prognosis of aSAH. The number of knots were determined by the lowest Akaike information criterion (AIC) value. Receiver operating characteristic (ROC) analysis was conducted to assess the predictive capability of HALP for clinical outcome. Net reclassification improvement (NRI) and integrated discrimination improvement (IDI) were employed to assess the incremental predictive value of HALP in conjunction with conventional risk factors. Delong test was used to analyze the difference of predictive ability between HALP, NLR, SIRI, and PNI. The robustness of correlation between HALP and functional outcome of aSAH was assessed through subgroup analysis. Finally, we also explored the relationship between HALP and postoperative complications. This study calculated the sample size based on Events Per Variable (EPV), a widely recognized metric in statistical analysis (9). The detailed method was presented in Supplementary material. All statistical analyses were performed using R version 4.4.0 Statistical Software.

## Results

### Patients enrollment

From January 2015 to September 2022, a total of 1,268 aSAH patients were included in the LongTEAM registry study. 106 patients were lost to follow up; 74 patients with a prior history of neurosurgical condition, and 340 patients with a missing laboratory test. After excluding 520 ineligible aSAH patients, 748 patients were enrolled in this study. The flow chart of patients enrollment was exhibited in Supplementary Figure 1. Among these patients, 210 aSAH patients (28.07%) had unfavorable functional outcomes.

### Baseline characteristics

The baseline characteristics analysis was shown in Table 1. 320 (42.78%) patients were male, 67 (8.96%) patients with Hunt Hess score 4–5, and 394 (52.67%) patients received surgical clipping. According to the baseline HALP levels (HALP < 30.286, 30.286 ≤ HALP ≤ 43.201, and HALP > 43.201), we categorized aSAH patients into three groups. Compared to patients with high HALP level, those with lower HALP levels tended to be females, with a greater percentage of WFNS grade 4–5 cases, with a greater percentage of Hunt Hess grade 4–5 cases, with a higher incidence of preoperative loss of consciousness, had a higher prevalence of postoperative MACE and pneumonia complications.

**Table 1:** Analysis of baseline characteristics.

Variables	Quartiles of HALP			*p*
	T1 (<30.286)	T2 (30.286–43.201)	T3 (>43.201)	
Number of patients	250	249	249	
Demographic data				
Age, years	55.00 (48.00,64.00)	57.00 (48.00,64.00)	54.00 (47.00,61.00)	0.057
Gender, *n* (%)				**0.045**
Female	156 (62.40)	144 (57.83)	128 (51.41)	
Male	94 (37.60)	105 (42.17)	121 (48.59)	
Rupture to admission, hours	24.00 (22.00,48.00)	24.00 (24.00,48.00)	24.00 (24.00,48.00)	0.164
Length of stay, day	12.00 (8.00,16.00)	11.00 (7.00,15.00)	11.00 (7.00,14.00)	0.238
Preoperative clinical status				
GCS score	13.00 (12.00,15.00)	15.00 (14.00,15.00)	15.00 (14.00,15.00)	**<0.001**
SEBES score 3–4, *n* (%)	110 (44.00)	106 (42.57)	115 (46.18)	0.716
mFS Score 3–4, *n* (%)	190 (76.00)	175 (70.28)	173 (69.48)	0.209
WFNS score 4–5, *n* (%)	60 (24.00)	40 (16.06)	39 (15.66)	**0.026**
Hunt Hess score 4–5, *n* (%)	39 (15.60)	13 (5.22)	15 (6.02)	**<0.001**
IVH, *n* (%)	179 (71.60)	162 (65.06)	162 (65.06)	0.199
Preoperative hydrocephalus, *n* (%)	109 (43.60)	103 (41.37)	90 (36.14)	0.220
Loss of consciousness, *n* (%)	88 (35.20)	65 (26.10)	61 (24.50)	**0.017**
Max diameter of aneurysm, mm	6.00 (4.50,8.52)	5.41 (4.00,8.00)	5.30 (4.00,8.00)	0.056
Treatment modality, *n* (%)				0.837
Endovascular interventional	116 (46.40)	115 (46.18)	121 (48.59)	
Surgical clipping	134 (53.60)	134 (53.82)	128 (51.41)	
Previous history				
Current smoking, *n* (%)	53 (21.20)	59 (23.69)	61 (24.50)	0.660
Current drinking, *n* (%)	40 (16.00)	44 (17.67)	41 (16.47)	0.875
Diabetes, *n* (%)	18 (7.20)	15 (6.02)	18 (7.23)	0.831
Hypertension, *n* (%)	148 (59.20)	142 (57.03)	130 (52.21)	0.273
History of heart disease, *n* (%)	57 (22.80)	41 (16.47)	39 (15.66)	0.078
History of antiplatelet, *n* (%)	2 (0.80)	0 (0.00)	1 (0.40)	0.777
History of anticoagulant, *n* (%)	13 (5.20)	8 (3.21)	11 (4.42)	0.543
In-hospital complications				
Postoperative MACE, *n* (%)	93 (37.20)	70 (28.11)	66 (26.51)	**0.020**
DCI, *n* (%)	79 (31.60)	61 (24.50)	62 (24.90)	0.133
Postoperative intracranial infection, *n* (%)	32 (12.80)	22 (8.84)	30 (12.05)	0.330
Postoperative stress ulcer, *n* (%)	59 (23.60)	44 (17.67)	47 (18.88)	0.217
Anemia, *n* (%)	104 (41.60)	88 (35.34)	81 (32.53)	0.098
Pneumonia, *n* (%)	113 (45.20)	90 (36.14)	75 (30.12)	**0.002**
DVT, *n* (%)	90 (36.00)	87 (34.94)	72 (28.92)	0.194
Laboratory test				
Preoperative Glu, mmol/L	7.70 (6.82,9.11)	7.47 (6.60,8.81)	7.35 (6.38,8.68)	**0.031**
Preoperative Crea, μmol/L	59.10 (45.70,63.50)	55.30 (47.00,66.60)	52.20 (47.30,69.30)	**0.026**
Preoperative ALT, IU/L	18.40 (13.00,23.88)	18.00 (13.30,26.00)	18.00 (13.00,28.00)	0.599
Preoperative AST, IU/L	21.00 (17.18,27.28)	20.50 (16.20,26.00)	20.00 (16.60,25.40)	0.162
Preoperative Tp, g/L	72.35 (68.32,76.35)	71.60 (68.00,75.60)	72.00 (68.70,76.00)	0.731
Preoperative Alb, g/L	42.20 (39.80,44.38)	42.00 (39.80,44.60)	42.40 (40.30,44.80)	0.245
Preoperative Wbc, 10^9^/L	12.70 (10.16,15.77)	12.27 (10.22,15.22)	11.83 (9.34,14.33)	**0.031**
Preoperative Mono, 10^9^/L	0.60 (0.27,0.85)	0.52 (0.40,0.64)	0.32 (0.18,0.48)	**<0.001**
Preoperative Neut, 10^9^/L	11.05 (8.33,13.47)	10.72 (8.71,13.64)	10.93 (8.01,14.51)	0.917
Preoperative Rbc, 10^9^/L	4.46 (4.15,4.79)	4.51 (4.22,4.80)	4.56 (4.25,4.85)	0.116
Preoperative Plt, 10^9^/L	200.00 (188.00,271.00)	192.00 (161.00,232.00)	187.00 (166.00,235.00)	0.233
Preoperative Hgb, g/L	140.00 (128.00,150.75)	152.00 (141.00,182.00)	155.00 (146.00,185.00)	**<0.001**
Preoperative Ly, 10^9^/L	0.78 (0.64,1.00)	1.14 (0.91,1.45)	1.66 (1.46,1.95)	**<0.001**
NLR	13.86 (9.40,18.78)	9.53 (6.99,12.71)	6.92 (4.44,9.11)	**<0.001**
PNI	46.15 (43.86,48.40)	47.95 (45.65,50.60)	51.20 (48.60,54.15)	**<0.001**
SIRI	4.99 (3.12,8.35)	3.88 (2.37,6.68)	3.50 (1.99,5.70)	**<0.001**
mRS, *n* (%)				**<0.001**
0–2	110 (44.00)	205 (82.33)	223 (89.56)	
3–6	140 (56.00)	44 (17.67)	26 (10.44)	

mFS, modified Fisher scale; SEBES, Subarachnoid Hemorrhage Early Brain Edema Score; IVH, intraventricular hemorrhage; GCS, Glasgow coma score; WFNS, World Federation of Neurological Societies; DCI, delayed cerebral ischemic; DVT, deep vein thrombosis; Glu, glucose; Crea, creatinine; ALT, alanine transaminase; AST, aspartate transaminase; Tp, total protein; Alb, albumin; PNI, prognostic nutritional index; NLR, neutrophil to lymphocyte ratio; SIRI, systemic inflammatory response index; Wbc, white blood cell; Ly, lymphocyte; Plt, platelet; Mono, monocyte; Neut, neutrophil; Rbc, red blood cell; Hgb, hemoglobin; mRS, modified Ranking score; MACE, major adverse cardiovascular events. Bold values: *p* < 0.05.

Additionally, patients with higher HALP were more likely to have lower levels of glucose, Crea, Wbc, monocyte, NLR, and SIRI, but higher levels of hemoglobin and lymphocyte. Furthermore, patients with high HALP levels demonstrated more favorable clinical outcomes.

### HALP levels and prognosis of aSAH patients

**Figure 1:**
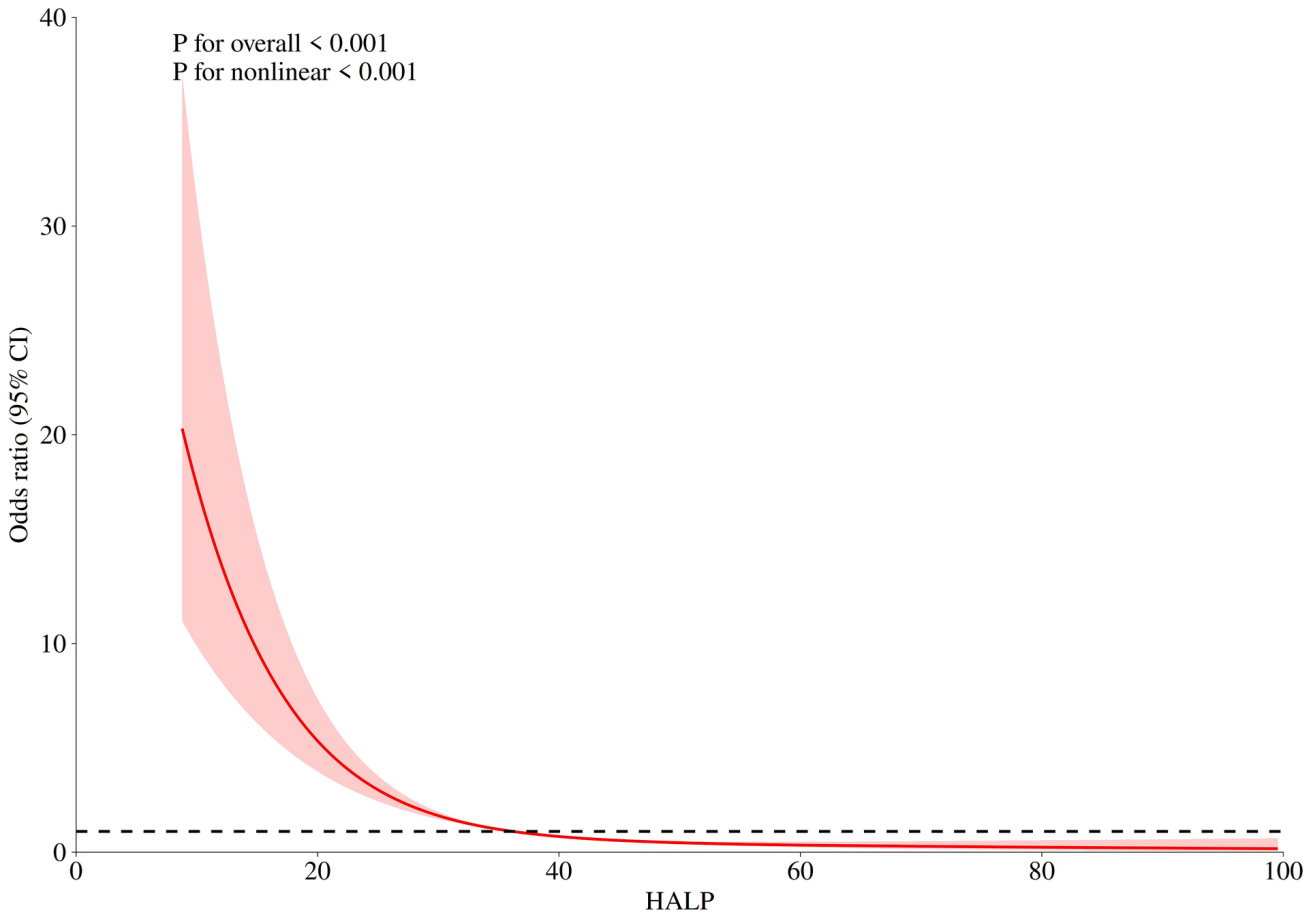
After adjusting for age, gender, Glasgow coma score, World Federation of Neurological Societies, Hunt Hess score, loss of consciousness, pneumonia, major adverse cardiovascular events, white blood cell, monocyte, glucose, and creatinine, the RCS analysis showed there was a non-linear relationship between HALP score levels and prognosis of aSAH.

The relationship between baseline HALP score levels and the risk of unfavorable functional outcome was presented in Table 2. In the fully adjusted model, the risk of unfavorable functional outcome decreased with each increment in HALP score levels Odds Ratio: 0.095, 95% Confidence Interval: 0.056–0.163, *p* value < 0.001. After adjusting for age, gender, loss of consciousness, pneumonia, preoperative GCS, MACE, preoperative WFNS, Wbc, preoperative Hunt Hess score, monocyte, glucose, and Crea, the RCS analysis showed there was an intriguing L-shaped relationship between HALP score levels and prognosis of aSAH (p for non-linear < 0.001), suggesting that as HALP score values increased, there was a progressive decrease in the risk of poor functional outcome (shown in Figure 1).

**Table 2:** The association between baseline HALP score levels and the risk of unfavorable outcome.

	The number of events (unfavorable outcome), *n* (%)	Crude model		Minimally adjusted model		Fully adjusted model	
		OR (95% CI)	*p*	OR (95% CI)	*p*	OR (95% CI)	*p*
HALP tertiles							
T1 (<30.286)	140 (18.71)	1.0(Ref)		1.0(Ref)		1.0(Ref)	
T2 (30.286–43.201)	44 (5.88)	0.165(0.109–0.250)	<0.001	0.156 (0.099–0.246)	<0.001	0.168 (0.107–0.265)	<0.001
T3 (>43.201)	26 (3.48)	0.093 (0.057–0.150)	<0.001	0.085(0.050–0.144)	<0.001	0.095(0.056–0.163)	<0.001

Crude model: age, gender. Minimally adjusted model: age, gender, Glasgow coma score, World Federation of Neurological Societies, Hunt Hess score, loss of consciousness, pneumonia, and major adverse cardiovascular events. Fully adjusted model: age, gender, Glasgow coma score, World Federation of Neurological Societies, Hunt Hess score, loss of consciousness, pneumonia, major adverse cardiovascular events, white blood cell, monocyte, glucose, and creatinine.

### The receiver operating curve of HALP score

The ROC curve indicated that the area under the curve (AUC) was 0.795. The optimal cut-off value was determined by the maximal Youden index (sensitivity + specificity−1). In this research, the optimal HALP cut-off value as a predictor was 30.456 (sensitivity: 79.6%, specificity: 67.6%). The result of ROC analysis identified that HALP had significant potential in predicting unfavorable functional outcomes in patients with aSAH (Table 3).

**Table 3:** The prediction ability of HALP for the unfavorable outcome of aSAH patients.

Characteristic	Area under the curve	Cut-off value	Sensitive	Specialty	Youden
HALP	0.795	30.456	0.796	0.676	0.472

### Incremental effect of HALP score in predicting unfavorable outcomes

**Figure 2:**
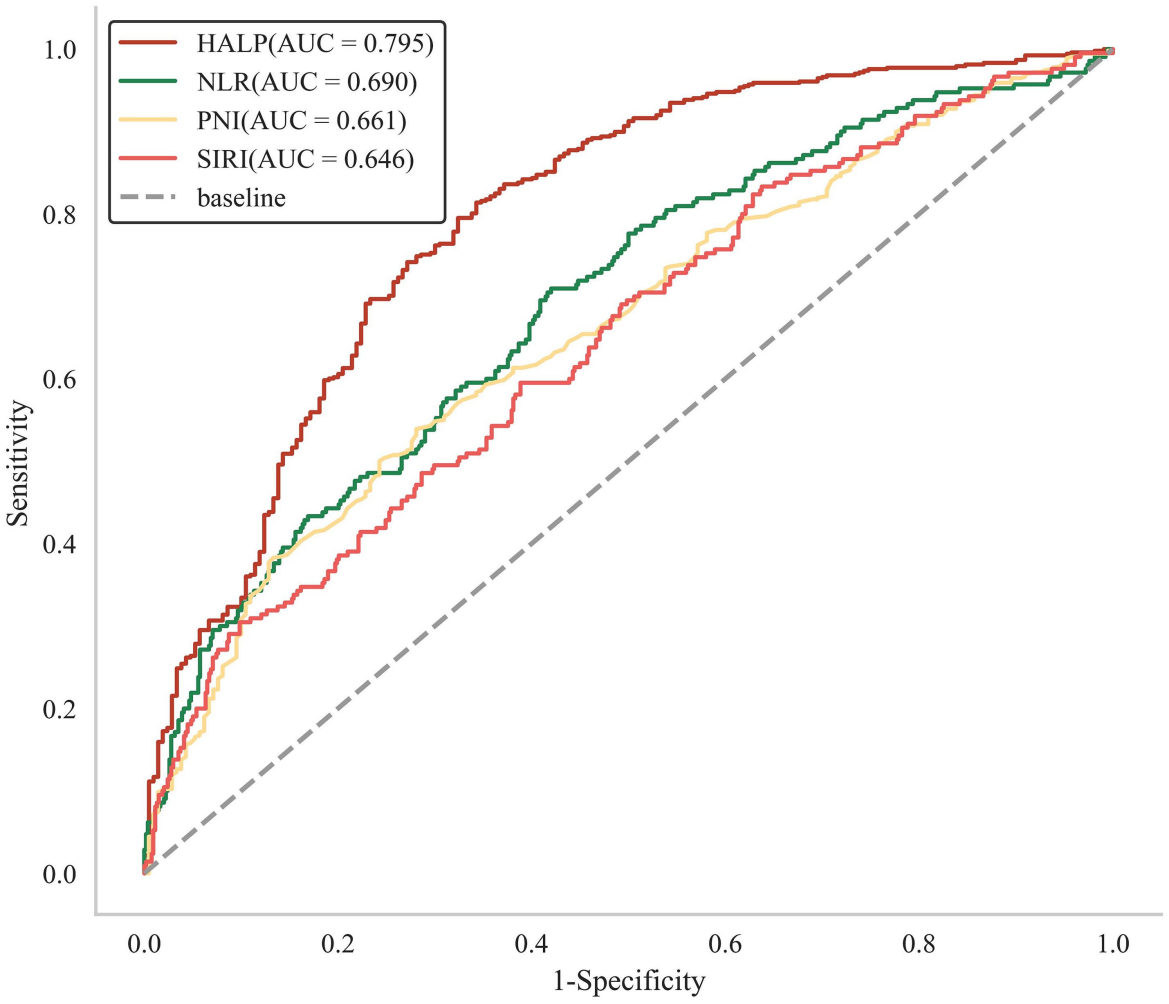
The ROC analysis of HALP, NLR, SIRI, and PNI.

To compare the predictive ability of HALP with other single inflammatory or nutritional indicators, we analyzed the ROC curve of NLR, SIRI, and PNI, as shown in Figure 2. The Delong test revealed that HALP score demonstrated a significantly greater predictive capability compared to the other three indicators (Supplementary Table 3). Furthermore, the NRI and IDI were calculated to determine whether adding the HALP score to TAPS mode enhances risk prediction for adverse prognosis in patients with aSAH. As presented in Table 4, the inclusion of the HALP score in the TAPS model (which comprises age, WFNS, mFS, Graeb score, white blood cell count, and surgical clipping) resulted in a notable enhancement in risk reclassification for unfavorable prognosis (NRI: 48.40%, *p* < 0.001; IDI: 19.09%, p < 0.001).

**Table 4:** Reclassification and discrimination statistics for unfavorable outcome of aSAH by HALP at baseline.

	Continuous NRI, %		IDI, %	
	Estimate (95% CI)	*p* value	Estimate (95% CI)	*p* value
SAH				
TAPS model	Ref		Ref	
TAPS model + HALP (continuous)	48.40(37.83–58.98)	<0.001	19.09(16.09–22.10)	<0.001

TAPS model: age>55 years old, WFNS grade of 4–5, mFS grade of 3–4, Graeb score of 5–12, white blood cell count>11.28 × 109 /L, and surgical clipping.

### The subgroup analysis of HALP

Table 5 shows the results of subgroup analysis for HALP. Based on the cut-off value of HALP score, we divided patients into low-HALP group (HALP < 30.456) and high-HALP group (HALP ≥ 30.456). The results of the analysis indicated that there were no notable interactions observed between the HALP score and the predetermined factors that were specified in the study. All *p*-values associated with these interactions were greater than 0.05, suggesting that any potential relationships between the HALP score and the identified factors were not statistically significant. This finding implied that the HALP score’s effects remain consistent across the various factors considered, reinforcing the robustness of the results presented in this research.

**Table 5:** The subgroup analysis of HALP score.

Variables	*n* (%)	<30.456	≥30.456	OR (95% CI)	*p*	*p* for interaction
Gender						0.447
Female	428 (57.22)	90/157	35/271	0.11 (0.07–0.18)	**<0.001**	
Male	320 (42.78)	52/96	33/224	0.15 (0.08–0.25)	**<0.001**	
Age						0.421
<65	591 (79.01)	105/193	48/398	0.11 (0.08–0.17)	**<0.001**	
≥65	157 (20.99)	37/60	20/97	0.16 (0.08–0.33)	**<0.001**	
Graeb score						0.240
0–4	695 (92.91)	127/232	56/463	0.11 (0.08–0.17)	**<0.001**	
5–12	53 (7.09)	15/21	12/32	0.24 (0.07–0.79)	**0.018**	
SEBES score						0.990
0–2	417 (55.75)	76/141	35/276	0.12 (0.08–0.20)	**<0.001**	
3–4	331 (44.25)	66/112	33/219	0.12 (0.07–0.21)	**<0.001**	
mFS Score						0.055
0–2	210 (28.07)	32/60	10/150	0.06 (0.03–0.14)	**<0.001**	
3–4	538 (71.93)	110/193	58/345	0.15 (0.10–0.23)	**<0.001**	
IVH						0.943
No	245 (32.75)	36/72	19/173	0.12 (0.06–0.24)	**<0.001**	
Yes	503 (67.25)	106/181	49/322	0.13 (0.08–0.19)	**<0.001**	
Preoperative hydrocephalus						0.646
No	446 (59.63)	80/143	39/303	0.12 (0.07–0.19)	**<0.001**	
Yes	302 (40.37)	62/110	29/192	0.14 (0.08–0.24)	**<0.001**	
WFNS score						0.537
1–3	609 (81.42)	90/192	41/417	0.12 (0.08–0.19)	**<0.001**	
4–5	139 (18.58)	52/61	27/78	0.09 (0.04–0.21)	**<0.001**	
Hunt Hess score						0.843
1–3	681 (91.04)	107/214	54/467	0.13 (0.09–0.19)	**<0.001**	
4–5	67 (8.96)	35/39	14/28	0.11 (0.03–0.41)	**<0.001**	
Rupture to admission						0.791
<24 h	178 (23.80)	44/69	21/109	0.14 (0.07–0.27)	**<0.001**	
≥24 h	570 (76.20)	98/184	47/386	0.12 (0.08–0.19)	**<0.001**	
Loss of consciousness						0.619
No	534 (71.39)	79/163	37/371	0.12 (0.07–0.19)	**<0.001**	
Yes	214 (28.61)	63/90	31/124	0.14 (0.08–0.26)	**<0.001**	
Current smoking						0.869
No	575 (76.87)	116/199	55/376	0.12 (0.08–0.18)	**<0.001**	
Yes	173 (23.13)	26/54	13/119	0.13 (0.06–0.29)	**<0.001**	
Current drinking						0.565
No	623 (83.29)	120/213	59/410	0.13 (0.09–0.19)	**<0.001**	
Yes	125 (16.71)	22/40	9/85	0.10 (0.04–0.25)	**<0.001**	
Diabetes						0.390
No	697 (93.18)	129/235	63/462	0.13 (0.09–0.19)	**<0.001**	
Yes	51 (6.82)	13/18	5/33	0.07 (0.02–0.28)	**<0.001**	
Hypertension						0.151
No	328 (43.85)	51/103	32/225	0.17 (0.10–0.29)	**<0.001**	
Yes	420 (56.15)	91/150	36/270	0.10 (0.06–0.16)	**<0.001**	
History of heart disease						0.448
No	611 (81.68)	103/195	55/416	0.14 (0.09–0.20)	**<0.001**	
Yes	137 (18.32)	39/58	13/79	0.10 (0.04–0.22)	**<0.001**	
History of antiplatelet						0.980
No	745 (99.60)	140/251	68/494	0.13 (0.09–0.18)	**<0.001**	
Yes	3 (0.40)	2/2	0/1	0.00 (0.00 - Inf)	1.000	
History of anticoagulant						0.578
No	716 (95.72)	136/240	67/476	0.13 (0.09–0.18)	**<0.001**	
Yes	32 (4.28)	6/13	1/19	0.06 (0.01–0.64)	**0.019**	

OR, Odds ratio; CI, Confidence interval. Bold values: *p* < 0.05.

### Association between HALP and in-hospital complications

**Figure 3:**
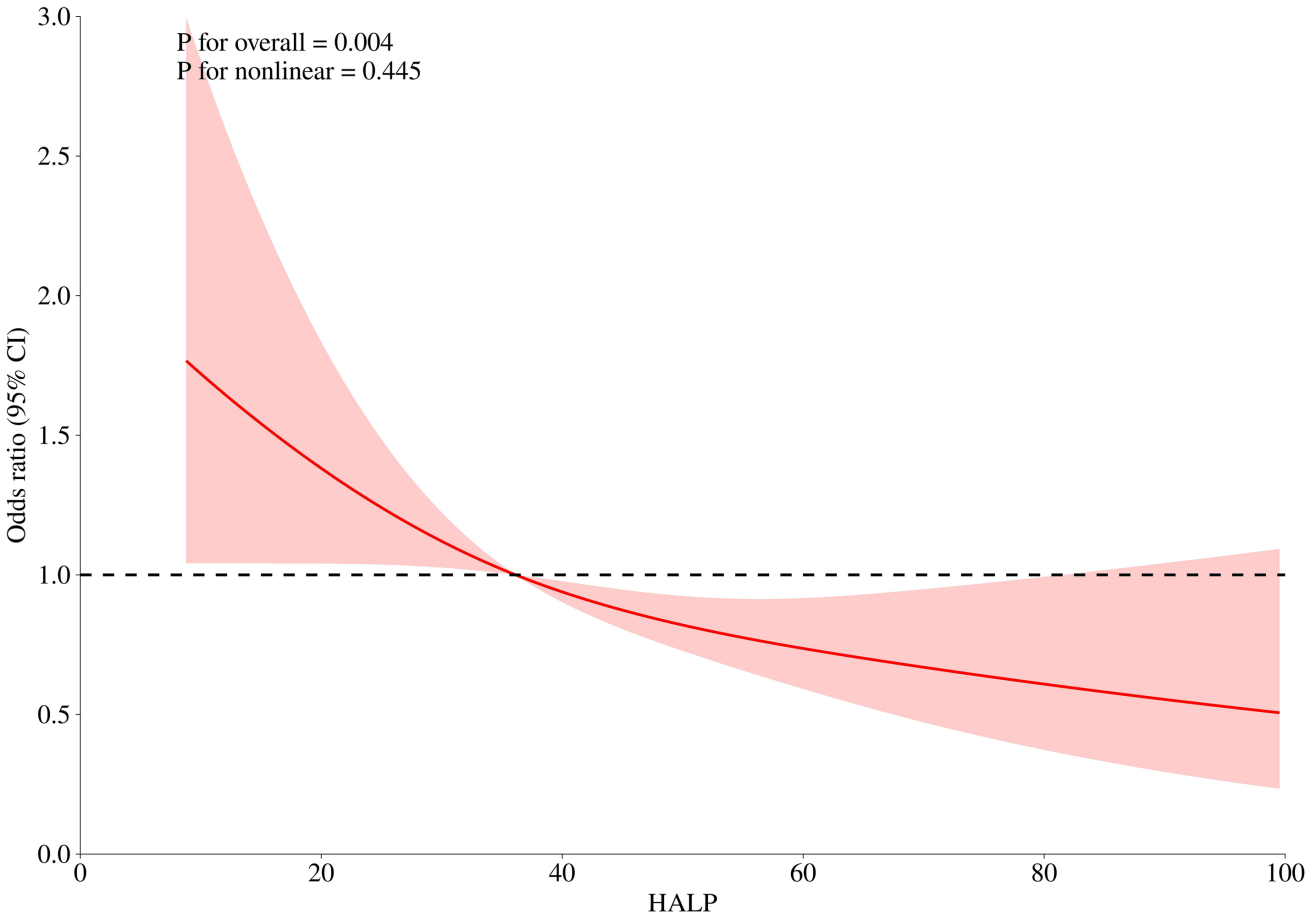
After adjusting for age, gender, Glasgow coma score, World Federation of Neurological Societies, Hunt Hess score, loss of consciousness, pneumonia, major adverse cardiovascular events, white blood cell, monocyte, glucose, and creatinine, the RCS analysis showed there was a linear relationship between HALP score levels and in-hospital pneumonia.

Among the 748 patients, pneumonia was identified as the most prevalent postoperative complication, occurring in 278 patients (37.16%), followed by anemia, which occurred in 273 patients (36.50%). After adjusting for age, gender, GCS, WFNS, Hunt Hess score, loss of consciousness, Wbc, monocyte, glucose, and Crea, multivariate logistic regression analysis revealed that patients in the highest HALP groups (>43.201) exhibited the lowest risk of pneumonia (OR: 0.586, 95% CI: 0.388–0.887, *p* = 0.012, as illustrated in Table 6). The RCS curve showed a significant linear relationship between pneumonia and HALP levels (*p* for non-linear = 0.445, p for overall = 0.004, as illustrated in Figure 3).

**Table 6:** The association between baseline HALP score levels and the risk of In-hospital complications.

Adjusted model		
In-hospital complications	OR (95% CI)	*p*
MACE		
T1 (<30.286)	1.0(Ref)	
T2 (30.286–43.201)	0.682 (0.456–1.019)	0.062
T3 (>43.201)	0.730 (0.477–1.177)	0.147
DCI		
T1 (<30.286)	1.0(Ref)	
T2 (30.286–43.201)	0.737 (0.489–1.112)	0.146
T3 (>43.201)	0.765 (0.497–1.177)	0.223
Intracranial infection		
T1 (<30.286)	1.0(Ref)	
T2 (30.286–43.201)	0.782 (0.428–1.427)	0.422
T3 (>43.201)	1.076 (0.595–1.944)	0.809
Stress ulcer		
T1 (<30.286)	1.0(Ref)	
T2 (30.286–43.201)	0.840 (0.524–1.348)	0.471
T3 (>43.201)	0.983 (0.601–1.608)	0.946
Anemia		
T1 (<30.286)	1.0(Ref)	
T2 (30.286–43.201)	0.902 (0.610–1.332)	0.603
T3 (>43.201)	0.826 (0.547–1.248)	0.364
Pneumonia		
T1 (<30.286)	1.0(Ref)	
T2 (30.286–43.201)	0.770 (0.520–1.141)	0.603
T3 (>43.201)	0.586 (0.388–0.887)	**0.012**
DVT		
T1 (<30.286)	1.0(Ref)	
T2 (30.286–43.201)	1.125 (0.746–1.696)	0.574
T3 (>43.201)	0.934 (0.607–1.436)	0.755

MACE, major adverse cardiovascular events; DCI, delayed cerebral ischemia; DVT, deep vein thrombosis. Adjusted model: age, gender, Glasgow coma score, World Federation of Neurological Societies, Hunt Hess score, loss of consciousness, pneumonia, major adverse cardiovascular events, white blood cell, monocyte, glucose, and creatinine.

## Discussion

In this retrospective study, the relationship between HALP and the clinical outcomes in patients with aSAH was investigated. After a thorough adjustment for various potential confounding variables that could affect the results, multivariate logistic regression revealed the risk of poor functional outcome decreased with each increase in HALP score levels. Interestingly, an intriguing L-shaped relationship between HALP and poor prognosis of aSAH patients was found through RCS analysis. Compared with other single inflammation or nutritional markers (NLR, SIRI, and PNI), HALP had a higher predictive ability and was able to significantly improve the predictive performance of TAPS model. Additionally, we also found that low HALP scores may increase the risk of postoperative pneumonia.

HALP, calculated using hemoglobin, albumin, lymphocyte, and platelet, has been identified as a novel index reflecting systemic inflammatory, immune and nutritional conditions. In 2015, Chen et al. devised HALP score and reported this index was an independent prognostic factor for gastric carcinoma (10). In recent years, an increasing number of studies have been conducted to explore the association between HALP score and prognosis of different diseases. According to Farag et al., HALP was demonstrated as a reliable prognostic factor in pancreatic cancer, biliary tract cancer, and bladder cancer (11). Furthermore, HALP had also been identified to be associated with the prognosis of numerous cardiovascular and peripheral vascular diseases (12, 13). Additionally, Zuo et al. also found that HALP was correlated with the prognosis and several complications of stroke patients (14–17). However, it remains unclear whether HALP could predict the clinical outcomes in patients with aSAH. To the best of our knowledge, this study is the first to reveal the association between HALP and the prognosis of aSAH, demonstrating this index has potential as a reliable predictive factor.

There are some potential pathological mechanisms to explain these findings. According to previous studies, early brain injury (EBI), an important pathological process occurring within the first 72 h after the onset of aSAH, is strongly associated with the prognosis of aSAH patients (18). During this period, the sudden increase of intracranial cerebral pressure (ICP) and decreased cerebral perfusion pressure (CPP) activate downstream pathways, including oxidative stress and inflammation, which lead to neurological function loss and significantly influence the prognosis (19). Ly were identified to be associated with the inflammatory response of EBI. When aneurysm ruptured, blood stimulated the central nervous system, activating the immune system, and leading to the release of a large number of lymphocytes. These lymphocytes may play an important role in antigen recognition, cell activation, and inhibiting immune-mediated cytotoxicity, which contributes to neurological protection and significantly mitigates the early brain damage (20). Therefore, a decreased number of lymphocytes suggests a diminished neuroprotective effect, which may exacerbate brain injury and result in unfavorable clinical outcomes. Moreover, the depletion of lymphocytes reflects an immunosuppression status after aSAH, increasing the risk of postoperative infection events (21). Platelet is also identified as an important inflammation mediator. Numerous clinical and experimental studies have found that elevated platelet levels are significantly associated with more severe early brain injury for aSAH patients (22). Yun et al. also reported that after aneurysm rupture, platelet would be activated, which increased the risk of micro-thrombosis, which adversely influenced the recovery of neurological function (23). Hemoglobin and albumin are both critical nutritional markers. Previous studies have identified aSAH patients with malnutrition are at higher risk of suffering worse prognosis (5, 24). More importantly, albumin also has been found to play an important role in inhibiting oxidative stress, thrombosis, and leukocyte adhesion, which could protect the neuron and promote neurological function recovery (17, 25). The HALP score is derived from these four blood markers mentioned above, which makes it a comprehensive and reliable index to evaluate inflammation-nutritional status and predict the prognosis of aSAH patients.

Notably, this research also compared the predictive ability of HALP score and three other individual inflammation or nutritional markers (SIRI, NLR, and PNI). All these three indices were considered to have potential in predicting the prognosis of aSAH patients. Several studies have demonstrated that aSAH patients with unfavorable clinical outcomes exhibit higher levels of SIRI and NLR at admission compared to those with favorable clinical outcomes (21, 26). Fan et al. reported that aSAH patients with low PNI levels had a significantly unfavorable prognosis at 180 days after discharge (5). In this research, Delong test revealed that the predictive power of HALP score was significantly superior to the other three indices, suggesting that HALP is more stable and suitable for applying as a reliable, effective and precise predictive tool in clinical management.

Another novel finding of our research is the significant linear relationship between postoperative pneumonia and HALP score. Postoperative pneumonia is a sever complication which may prolong hospitalization and increase mortality in aSAH patients (27). Previous studies have demonstrated that some inflammation-nutrition markers, including neutrophil to albumin ratio, neutrophil to lymphocyte ratio, and SIRI, were associated with pneumonia (27–29). Additionally, in 2024, Wang et al. developed a machine learning model for predicting pneumonia after aSAH (30). Our research identifies high HALP decreases the risk of pneumonia, which may provide a new management target for early detecting and preventing postoperative pneumonia.

Our study also had several limitations. First, this is a single-center retrospective study, which may induce unavoidable bias. Second, the HALP score was only obtained within 24 h of admission after aSAH onset, and the dynamic variation of the HALP score at various stages of aSAH had not been investigated. Third, patients were followed up at 90 days after discharge. We believe a longer follow-up is needed to provide more robust data on the predictive value of the HALP score. Furthermore, due to the unavailability of detailed patient survival data, survival analysis could not be performed. Fourth, although our study period encompassed the COVID-19 pandemic, all emergency patients tested negative for SARS-CoV-2 in both preoperative and in-hospital nucleic acid tests, indicating that no COVID-19 cases were included in our analysis. Further research is warranted to investigate the association between the HALP score and the prognosis of aSAH patients with concurrent COVID-19 or other infectious diseases. Finally, although the multivariate logistic regression model adjusts for several important factors, some confounders that may influence clinical outcomes could still be present. Potential confounding factors, such as comorbid conditions, medications, and lifestyle factors were collected in our study. Therefore, a multi-center prospective study with a large sample size is required to validate our findings.

## Conclusion

This study demonstrates that a low preoperative HALP score may increase the risk of poor functional outcome and postoperative pneumonia in aSAH patients. Given that HALP score may contribute to identifying aSAH patients at high risk for poor prognosis, these findings hold significant clinical relevance. More prospective studies are needed to confirm this finding.

## Data Availability

The raw data supporting the conclusions of this article will be made available by the authors, without undue reservation.
