# Supplementary material for: Association between HALP score and clinical outcome in patients with aneurysmal subarachnoid hemorrhage: insights from a large cohort study
Source: Front Neurol. 2025 Sep 5;16:1667743. doi: 10.3389/fneur.2025.1667743 (PMC12446961; doi:10.3389/fneur.2025.1667743)
Supplement: Supplementary file 1 [file Table_1.DOCX]

This study calculated the sample size based on Events Per Variable (EPV), a widely recognized metric in statistical analysis. The incidence of adverse clinical outcomes in this study was 0.28. Considering that 12 variables needed to be included in the multivariate logistic regression analysis and the EPV was set to 10, the required sample size was calculated using the following formula: Sample size = (Number of variables × EPV) / Incidence of adverse outcomes. The final calculation indicated that 428 patients needed to be included. Our study enrolled 748 patients, which meets the statistical power requirement.

Supplemental Figure 1 The flow chart of patients enrollment


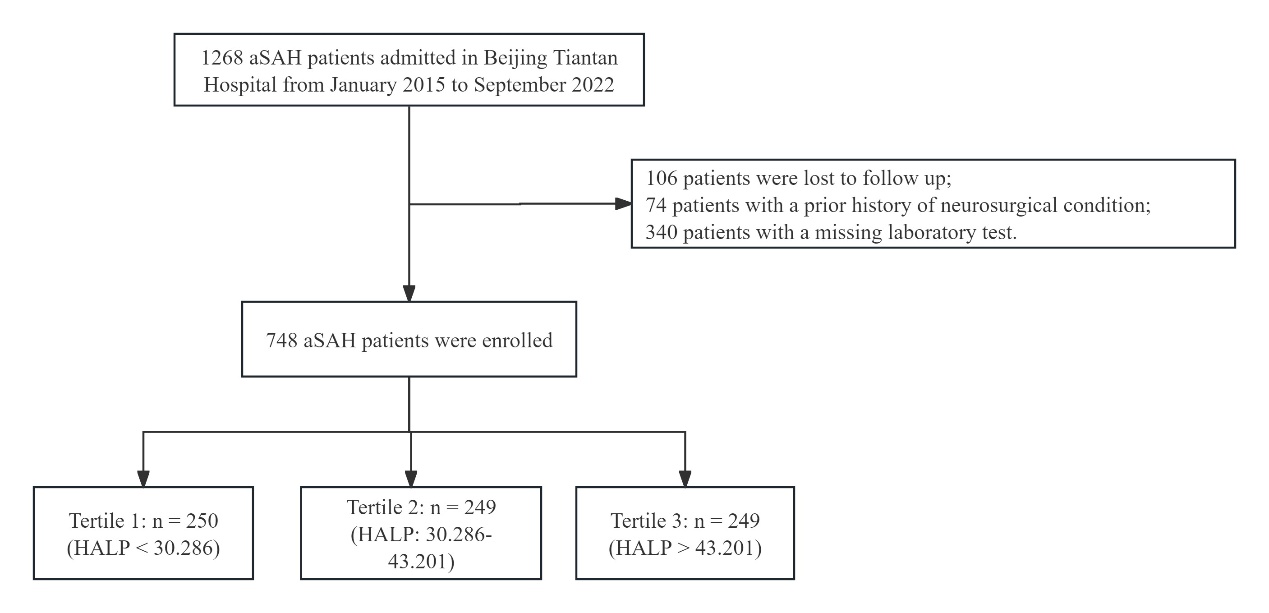


**S**upplemental Table 1. Definitions of in-hospital complications.

| In-hospital complications | Definitions |
| --- | --- |
| Delayed cerebral ischemia | New focal neurological deficit or global neurological deterioration (a drop of ≥ 2 points on the Glasgow Coma Scale [GCS]) lasting > 2 hours, after exclusion of intracranial hemorrhage, hydrocephalus, seizures, metabolic derangements, and infection, with or without radiological signs of cerebral vasospasm. |
| Intracranial infection | (1) The patient had clinical manifestations of various intracranial infections such as postoperative fever, headache, or neck stiffness. (2) The patient’s cerebrospinal fluid test showed inflammatory index changes, which met one of the following: white blood cell count > 0.01 × 10^9^ /L; cerebrospinal fluid protein > 4.50 g/L; cerebrospinal fluid glucose < 2.50 mmol/L. (3) The peripheral blood white blood cell count was > 10 × 10^9^ /L. |
| Major adverse cardiac event | The composite of total death; myocardial infarction; stroke, hospitalization because of heart failure; and revascularization, including percutaneous coronary intervention, and coronary artery bypass graft. |
| Stress ulcer bleeding | No previous history of peptic ulcer or peptic hemorrhage, and after treatment for aSAH, patients were tested positive by fecal occult blood test. |
| Anemia | In men, a hemoglobin of less than 130 to 140 g/L (13 to 14 g/dL); in women, it is less than 120 to 130 g/L (12 to 13 g/dL). |
| Pneumonia | Description of clinical indications or positive chest radiograph. |
| Deep vein thrombosis | Clinical diagnosis of deep vein thrombosis supported by ultrasound or venography. |

| Supplemental Table 2. Laboratory examination items reference ranges. | | |
| --- | --- | --- |
| Items | Reference range | Units |
| WBC | 3.5-9.5 | 10^9^/L |
| Hgb | 120-160 | g/L |
| Plt | 100-300 | 10^9^/L |
| Crea | 59-104 | μmol/L |
| ALT | 0-41.0 | IU/L |
| AST | 0-42.0 | IU/L |
| Total Protein | 60.0-80.0 | g/L |
| Albumin | 35.0-55.0 | g/L |
| WBC white blood cell; AST aspartate aminotransferase; ALT alanine aminotransferase; Plt platelet; Crea, Creatinine . | | |

| HALP vs NLR | |
| --- | --- |
| z statistic | 5.276 |
| Significance level | P < 0.001 |
| HALP vs PNI | |
| z statistic | 6.282 |
| Significance level | P < 0.001 |
| HALP vs SIRI | |
| z statistic | 5.757 |
| Significance level | P < 0.001 |

**Supplemental Table 3**. Delong Test for AUC of HALP, NLR, SIRI, and PNI
